# Interdisciplinary Treatment of Breast Cancer After Mastectomy With Autologous Breast Reconstruction Using Abdominal Free Flaps in a University Teaching Hospital—A Standardized and Safe Procedure

**DOI:** 10.3389/fonc.2020.00177

**Published:** 2020-03-05

**Authors:** Dominik Steiner, Raymund E. Horch, Ingo Ludolph, Marweh Schmitz, Justus P. Beier, Andreas Arkudas

**Affiliations:** ^1^Department of Plastic and Hand Surgery, University Hospital of Erlangen, Friedrich-Alexander University of Erlangen-Nuernberg, Erlangen, Germany; ^2^Department of Plastic Surgery, Hand Surgery, Burn Center, University Hospital RWTH Aachen, Aachen, Germany

**Keywords:** breast reconstruction, ms-TRAM, DIEP, CTA, venous coupler, interdisciplinary

## Abstract

**Background:** Breast cancer is the most common malignancy in women. The interdisciplinary treatment is based on the histological tumor type, the TNM classification, and the patient's wishes. Following tumor resection and (neo-) adjuvant therapy strategies, breast reconstruction represents the final step in the individual interdisciplinary treatment plan. Although manifold flaps have been described, abdominal free flaps, such as the deep inferior epigastric artery perforator (DIEP) or the muscle-sparing transverse rectus abdominis myocutaneous (ms-TRAM) flap, are the current gold standard for autologous breast reconstruction. This retrospective study focuses on the safety of autologous breast reconstruction upon mastectomy using abdominal free flaps.

**Methods:** From April 2012 until December 2018, 193 women received 217 abdominal free flaps for autologous breast reconstruction at the University Hospital of Erlangen. For perforator mapping, we performed computed tomography angiography (CTA). Venous anastomosis was standardized using a ring pin coupler system, and flap perfusion was assessed with fluorescence angiography. A retrospective analysis was performed based on medical records, the surgery report, and follow-up of outpatient course.

**Results:** In most cases, autologous breast reconstruction was performed as a secondary reconstructive procedure after mastectomy and radiotherapy. In total, 132 ms1-TRAM, 23 ms2-TRAM, and 62 DIEP flaps were performed with 21 major complications (10%) during hospital stay including five free flap losses (2.3%). In all cases of free flap loss, we found an arterial thrombosis as the main cause. In 24 patients a bilateral breast reconstruction was performed without free flap loss. The majority of free flaps (96.7%) did not need additional supercharging or turbocharging to improve venous outflow. Median venous coupler size was 2.5 mm (range, 1.5–3.5 mm).

**Conclusion:** Using CTA, intraoperative fluorescence angiography, titanized hernia meshes for rectus sheath reconstruction, and venous coupler systems, autologous breast reconstruction with DIEP or ms-TRAM free flaps is a safe and standardized procedure in high-volume microsurgery centers.

## Introduction

Breast cancer is the most commonly diagnosed cancer type in women (24.2%) with an annual incidence and mortality of 11.6 and 15%, respectively (1). As previously reported, autologous breast reconstruction upon mastectomy improves quality of life and is superior to alloplastic methods (2). In the past 40 years, autologous breast reconstruction went through a consequent development. Starting with the rediscovery and popularization of the pedicled latissimus dorsi flap for thoracic wall defects by Olivari in the early 1970s, the invention of muscle-sparing free TRAM flaps by Holmström and later the description of the pedicled transverse rectus abdominis myocutaneous (TRAM) flap by Hartrampf et al. (3) were the next evolutionary steps (4). Nowadays, the reconstructive surgeon can rely on a broad spectrum of free flaps such as the transverse myocutaneous gracilis, superior/inferior gluteal artery perforator, or abdominal free flaps (5). The later ones experienced a further refinement starting from the TRAM over the muscle-sparing variants (ms-TRAM) to the deep inferior epigastric artery perforator (DIEP) flap. Because of their low donor site morbidity, ms-TRAM and DIEP flaps represent the gold standard in autologous breast reconstruction (6–9).

In the past years, many high-volume microsurgery centers have established and improved several methods regarding perforator mapping, quantitative flap perfusion assessment, or donor site morbidity reduction, to make autologous breast reconstruction a standardized and safe procedure. Unlike centers, where one or two surgeons perform breast reconstruction with abdominal free flaps, we tried to answer the question if in an academic university hospital setting with a high number of various surgeons and teaching tasks this procedure is still safe and if there is a difference to published series from single surgeon's experiences.

In this retrospective analysis, we therefore analyzed the various factors that might be relevant in autologous breast reconstruction using abdominal free flaps, computed tomography angiography (CTA) for perforator mapping, venous coupler devices, intraoperative fluorescence angiography, and rectus sheath reconstruction with titanized hernia meshes.

## Methods

**Figure 1:**
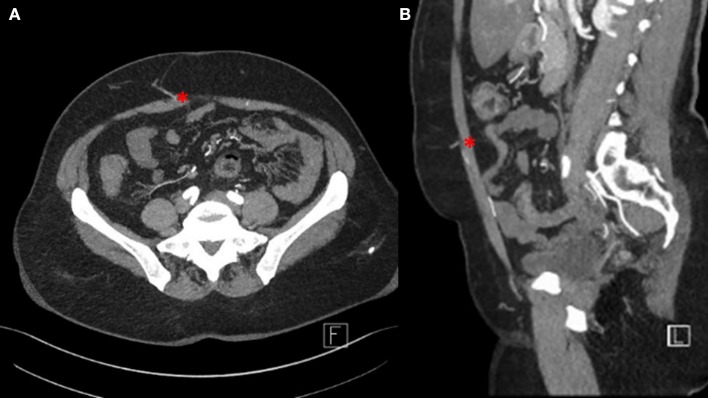
Perforator mapping using computed tomographic angiography (CTA) of the abdomen. **(A)** Transversal view. **(B)** Sagittal view.

Prior to surgery, all patients underwent CTA of the abdomen for perforator mapping (Figure 1). Based on the perforator anatomy (size, course, number), the patients were elected for autologous breast reconstruction with either DIEP or ms-TRAM free flaps. Moreover, only patients suitable for free tissue transfer (without morbid obesity or coagulation disorders) and with anesthesiologic acceptable risks underwent autologous breast reconstruction. No further exclusion criteria were defined. Seven senior surgeons performed autologous breast reconstruction in a 2-team approach. Flap harvest and vessel preparation occurred simultaneously. Flap harvest was performed by one of the senior surgeons. The internal mammary artery and vein were chosen as the primary recipient vessels. Mostly, a resident prepared the recipient vessels and assisted the senior surgeon during the microvascular anastomosis. Venous anastomoses were performed using a ring-pin coupler system from Synovis (St. Paul, MN, USA). Arterial anastomoses were hand-sewn with Ethilon 8-0 (Ethicon Inc., Somerville, NJ, USA). As previously reported, flap perfusion was assessed with fluorescence angiography using the SPY Elite Imaging System (Stryker, Kalamazoo, MI, USA) (10, 11). In case of fragile and/or recurrent thrombotic internal mammary artery, the vascular surgeons performed bypass extensions using the subclavian or thoracoacromial artery and a vein graft. In terms of primary breast reconstruction (*n* = 8), five prophylactic mastectomies and three mastectomies upon breast conserving therapy were performed. Rectus sheath closure or reconstruction and abdominal wound closure were performed using a TiMESH graft (pfm medical ag, Köln, Germany) in all cases. In case of postoperative hernia, four patients underwent laparoscopic (*n* = 3) or open (*n* = 1) hernia repair. For the retrospective analysis, we reviewed the complete medical charts and surgery reports. We used GraphPad Prism 7 (GraphPad Software, San Diego, CA, USA) for statistical analysis. Normal distribution was assessed with Shapiro-Wilk test. Further analysis was performed with multiple comparisons (using Tukey or Kruskal-Wallis test), Mann-Whitney *U* test, and Fisher exact test. *p* ≤ 0.05 are considered as statistically significant. This study was approved by the ethical review committee of the Friedrich-Alexander-University of Erlangen-Nuremberg (AZ 291_19 Bc).

## Results

**Figure 2:**
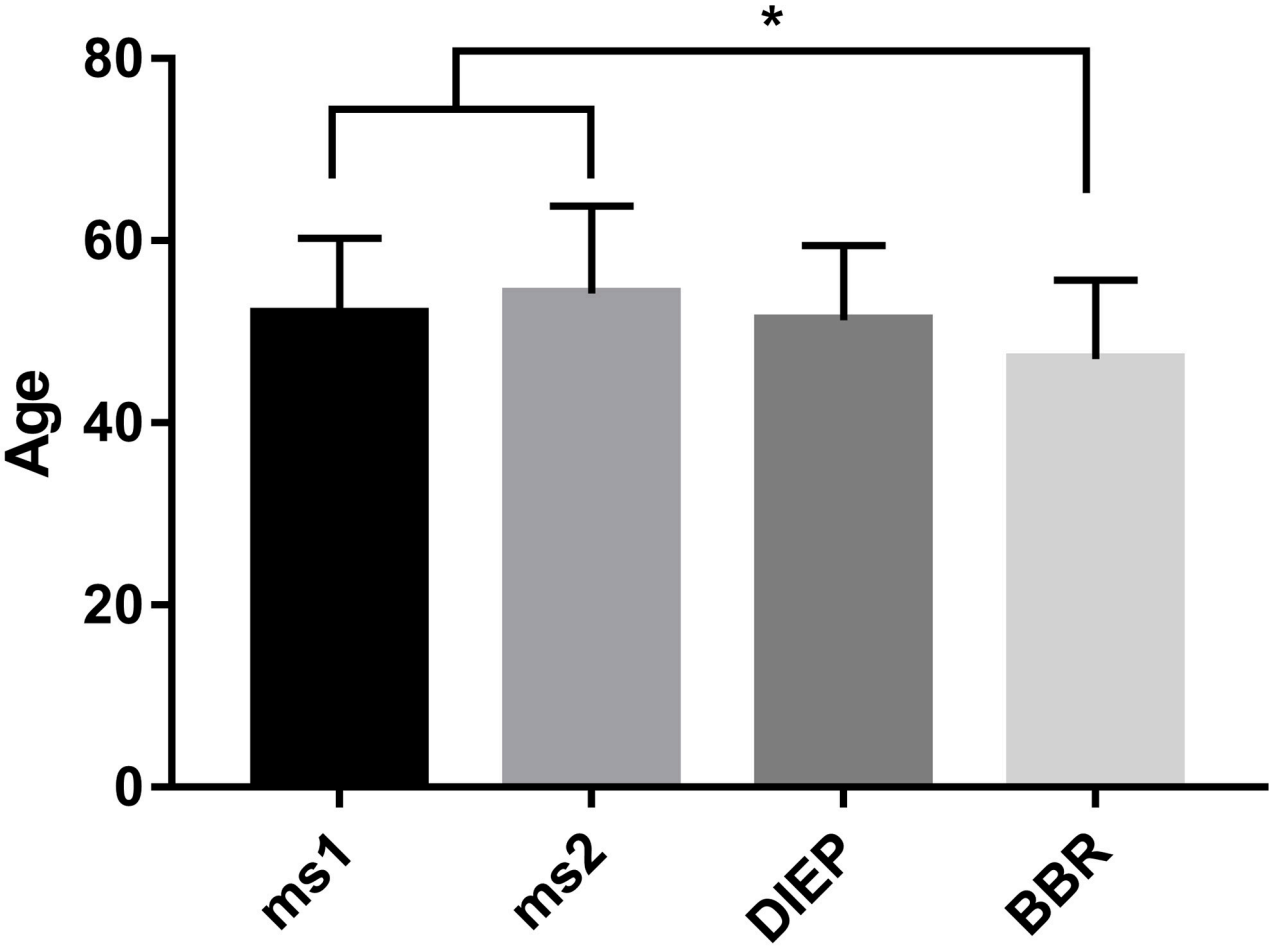
Statistically significant younger patients underwent bilateral breast reconstruction (BBR) compared to unilateral breast reconstruction using muscle-sparing transverse rectus abdominis myocutaneous flap. *p ≤ 0.05.

**Figure 3:**
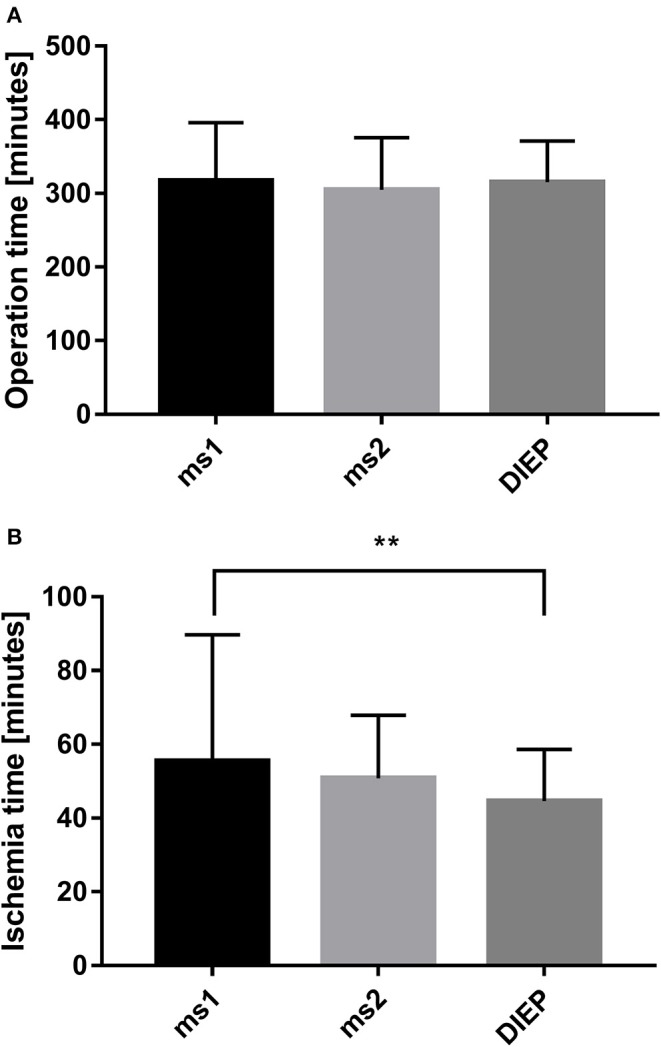
Operation time of the different flap types in unilateral breast reconstruction did not demonstrate statistically significant differences **(A)**. Comparing the ischemia time with the flap type, we found the shortest ischemia time in the deep inferior epigastric artery perforator (DIEP) group **(B)**. **p ≤ 0.01.

**Figure 4:**
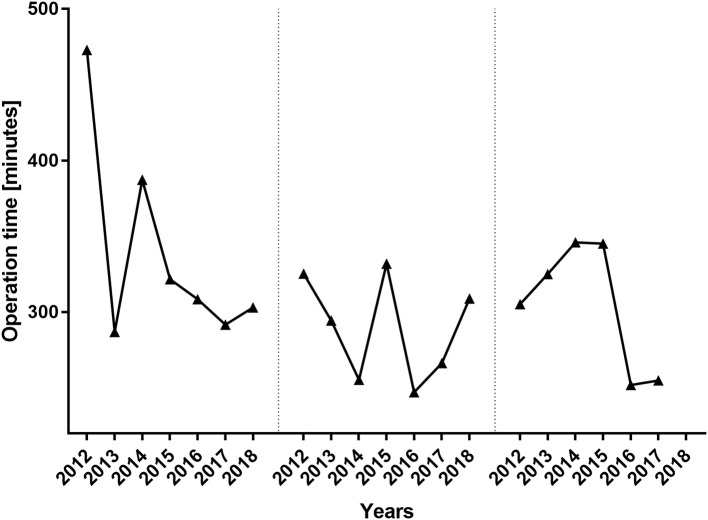
Operation time per surgeon from 2012 until 2018. The operation times of the three major surgeons who performed 88% of the unilateral breast reconstructions are depicted. Despite the years 2012 and 2014, the operation times did not differ significantly between the three senior surgeons.

During the period between 2012 and 2018, 193 women received 217 abdominal free flaps for autologous breast reconstruction at the Department of Plastic and Hand Surgery of the University Hospital of Erlangen. Thereof 24 patients underwent bilateral breast reconstruction (BBR). Average follow-up time was 41.2 months. Mostly, the patients were elected for secondary breast reconstruction (96%). Mean age of the patients was 50.5 ± 8.15 years. Compared to the patients receiving a unilateral ms-TRAM free flap, we found statistically significant younger patients in the bilateral reconstruction group (47.42 ± 16.04, *p* ≤ 0.05) (Figure 2). Most patients (*n* = 122) displayed a body mass index (BMI) of <30 kg/m^2^ in contrast to 50 women with a BMI of >30 kg/m^2^; 114 patients (59%) were irradiated, and 55 patients (28.5%) received chemotherapy. In total, 132 ms1-TRAM (60.8%), 23 ms2-TRAM (10.6%), and 62 DIEP flaps (28.6%) were used. Mean operation time for unilateral breast reconstruction was 315.18 ± 32.47 min without statistically significant differences between ms-TRAM and DIEP flaps (Figure 3). Obviously, the mean operation time was longer in the bilateral reconstruction group (455.7 ± 99.2; *p* ≤ 0.001). Mean flap ischemia time was 52.2 ± 29.4 min with the shortest ischemia times in the DIEP group (44.6 ± 14; *p* ≤ 0.01) (Figure 3). Next, we compared the operation time from 2012 until 2018. Operation time was defined as the interval between the first skin incision until complete wound closure. We analyzed the operation times from three senior surgeons who performed 149 of 169 unilateral breast reconstructions (88%). In this context, each senior surgeon reached a relatively stable minimum operation time (range, 247–309) after 5 years (Figure 4).

In order to improve venous outflow, additional turbocharging or supercharging was necessary in 2.3 and 1%, respectively. For turbocharging, additional anastomoses were performed between the superficial epigastric inferior and the deep inferior epigastric vein (*n* = 5). In case of supercharging, the ipsilateral cephalic vein was used additionally to the internal mammary vein (*n* = 2).

Most commonly, DIEP flaps required additional turbocharging or supercharging (*n* = 4) followed by ms1-TRAM flaps (*n* = 3). Flap characteristics are shown in Tables 1, 2.

**Table 1:** Flap characteristics in unilateral breast reconstruction.

	**ms1**	**ms2**	**DIEP**
Number	100	16	53
Primary reconstruction	2	1	0
Secondary reconstruction	98	15	53
Turbocharging	3	0	2
Supercharging	0	0	2
Complications	15	3	3
Flap loss	3	1	1
Radiation therapy	63	11	31
Chemotherapy	24	4	16

**Table 2:** Flap characteristics in bilateral breast reconstruction (BBR).

	**ms1**	**ms2**	**DIEP**
Number	32	7	9
Primary reconstruction	0	1	0
Secondary reconstruction	32	6	9
Turbocharging	2	0	0
Supercharging	1	0	1
Complications	0	0	1
Flap loss	0	0	0
Radiation therapy	8		
Chemotherapy	11		

*DIEP, deep inferior epigastric artery perforator*.

Mostly, the internal mammary artery was used for arterial anastomosis (98.2%). Because of recurrent intraoperative thrombosis, a vascular bypass using the subclavian (*n* = 2) or thoracoacromial (*n* = 2) artery and a vein graft was necessary in four patients. In two patients, the cephalic vein was used because of insufficient venous drainage of the internal mammary vein.

**Figure 5:**
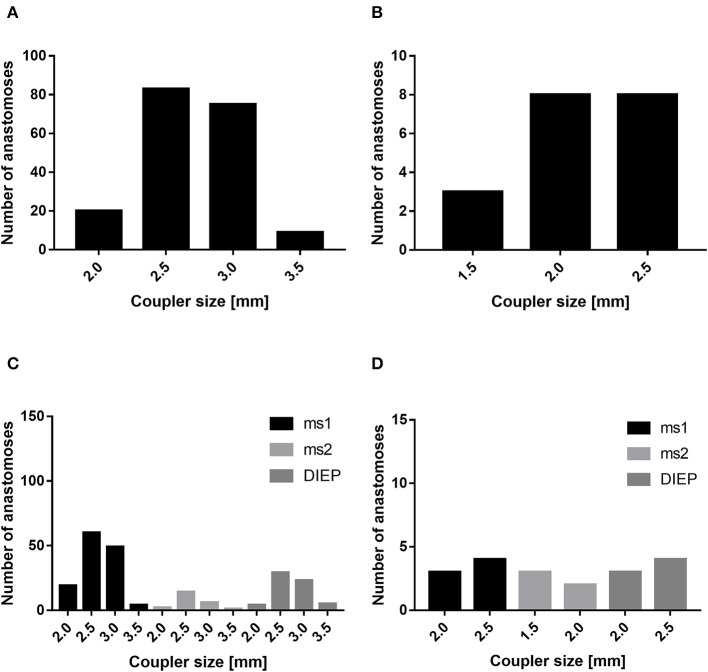
Mostly, the coupler diameter varied between 2.5 and 3.0 mm **(A)**. If a second venous anastomosis was performed, the coupler size varied between 1.5 and 2.5 mm **(B)**. The coupler size did not differ between muscle-sparing transverse rectus abdominis myocutaneous (ms-TRAM) and deep inferior epigastric artery perforator. (DIEP) flaps **(C)**. In case of a second venous anastomosis, the coupler size was smaller in the ms2-TRAM group **(D)**.

In our patient cohort, the internal mammary artery was mostly accompanied by one vein (81%). If one venous anastomosis was performed, the coupler diameter varied between 2.5 and 3.0 mm (48.8 and 34.6%, respectively). In 22 patients, a secondary venous anastomosis was performed with a median coupler diameter of 2.0 mm (range, 1.5–2.5 mm) (Figures 5A,B). Comparing the diameter of the venous coupler device, we were able to prove smaller diameters of the first venous anastomosis if a second anastomosis was additionally performed (2.55 ± 0.342 vs. 2.7 ± 0.371 mm; *p* ≤ 0.05). Considering the coupler size for the first venous anastomosis, the diameter varied between 2 and 3.5 mm without statistically significant differences between ms1-TRAM, ms2-TRAM, or DIEP flaps. Regarding the coupler size for the second venous anastomosis, ms2-TRAM group displayed smaller coupler diameters (range, 1.5–2.0 mm) compared to the ms1-TRAM or DIEP group (range, 2.0–2.5 mm) (Figures 5C,D). In case of secondary venous anastomosis, the medial and lateral internal mammary vein (*n* = 14) or the cranial and the caudal part of a solitary internal mammary vein was used for anastomosis (*n* = 8).

Considering the need of an additional charging procedure (turbocharging or supercharging; *n* = 7), we did not find a correlation between BMI of <30 kg/m^2^ (*p* = 0.3230), radiation therapy (*p* > 0.9999), or flap choice (muscle-sparing TRAM vs. DIEP; *p* = 0.2292).

Twenty-one major complications during hospital stay were registered. In most cases, secondary bleeding or hematoma (*n* = 6) was the main reason for revision surgery. Venous congestion (*n* = 3) and arterial thrombosis (*n* = 4) were the second leading cause for flap revision. Other major complications were umbilicus necrosis (*n* = 4), wound infection (*n* = 1), and abdominal wound healing disorder (*n* = 2). Five free flap losses were found (2.3%). In four patients, an arterial thrombosis was the cause for flap loss, whereas in the fifth case a disorder of cutaneous microcirculation led to partial flap loss (*n* = 1). In three of four cases, in which arterial reconstruction was necessary using the subclavian or thoracoacromial artery and a vein graft, flap loss was observed in the postoperative period. Regarding major complications during hospital stay associated with arterial or venous thrombosis, we did not find a correlation with BMI of >30 kg/m^2^ (*p* > 0.9999) or radiation therapy (*p* = 0.4716).

In four patients (2%), we found abdominal hernia in the postoperative aftercare (11–30 months after free flap harvest) requiring hernia repair. In these cases, a ms1-TRAM abdominal free flap was used for breast reconstruction with a tension-free anatomical reconstruction of the anterior rectus sheath using titanized hernia meshes in sublay technique.

## Discussion

Free microsurgical breast reconstruction with autologous tissue remains the gold standard in modern therapeutic strategies following mastectomy and especially when irradiation was performed during cancer treatment. Other techniques, such as tissue engineering and regenerative medicine, also including the prospect of three-dimensional printing, seem promising but have not reached the clinical applicability so far (12–14). In this retrospective study, we analyzed the outcome of 217 abdominal free flaps for autologous breast reconstruction in 193 patients with respect to the multisurgeon teaching aspect in a university hospital. Herein, we describe our approach including preoperative CTA, venous coupler systems, rectus sheath reconstruction, and intraoperative fluorescence angiography to assess flap perfusion, as well as the inclusion of other medical disciplines such as radiologists, gynecologists, and vascular or general surgeons. Nearly all women underwent secondary breast reconstruction. In 4%, our patients underwent primary breast reconstruction. In these selected cases, the oncological gynecologists performed mastectomy prior to autologous breast reconstruction.

For perforator as well as pedicle mapping, a preoperative CTA was performed. Of course, the preoperative use of CTA might display a certain risk of selection bias concerning the low major complication rate in our series. On the other hand, consistent with the pertinent literature, we believe that CTA enhances the inclusion of appropriate perforators while reducing the operation time and donor site morbidity (15–19). Computed tomography angiography does not only offer the possibility to visualize the architecture of the deep inferior artery and its perforators but also detects anomalous connections between the superficial and deep inferior epigastric venous system (20). The latter ones can affect venous outflow requiring additional charging procedures (supercharging or turbocharging) or the use of another flap type to prevent flap failure (21).

In 1962, Nakayama introduced the first vascular coupler system (22). From then on, the devices were consequently further developed in order to improve their efficacy and safety. Since 2009, our clinic uses venous coupler systems for free tissue transfer. In our cohort, median coupler size was 2.5 mm, without any statistically significant differences between ms1-TRAM, ms2-TRAM, and DIEP flaps. In accordance with other groups, the coupler size varied between 2.5 and 3.0 mm for most abdominal free flaps (23–26). We believe that venous coupler systems reduce the operation time, flap ischemia, venous thrombosis, and consequently flap failure. In the pertinent literature, venous thrombosis rate using venous coupler devices ranges between 0 and 4% (23–25, 27–30). In our cohort, we encountered three cases (1.4%) in which venous congestion was the main cause for revision surgery. In one case, venous congestion occurred intraoperatively during BBR, due to insufficient venous flow in the ipsilateral internal mammary vein after thrombosis of a subclavian port system in the medical history. We solved this problem using a venous crossover bypass to the contralateral caudal internal mammary vein (31). In the other two cases, a postoperative venous congestion occurred. In these two cases, venous coupler size was 2.5 mm. Bearing in mind that smaller diameters of the coupler device can affect venous congestion, we believe that a coupler size of <2.5 mm is associated with a higher risk of venous congestion (26). Supercharging and turbocharging procedures were necessary in 1 and 2.3%, respectively.

Although other risk factors, such as radiotherapy or obesity, are discussed in the literature, we could not prove an influence of previous radiation therapy or a BMI of >30 kg/m^2^ on vessel-associated complications (32–35). Furthermore, flap failure was not associated with venous thrombosis underlining the superiority of venous coupler systems compared to hand-sewn anastomoses (23, 30, 36). As a preliminary finding, the combination of venous coupler anastomosis and preoperative CTA is a valuable tool to enhance the safety of autologous breast reconstruction using abdominal free flaps (37).

In most cases, the internal mammary vessels were used as recipient vessels. Because of fragile and/or recurrent thrombotic internal mammary artery, arterial reconstruction was necessary in four patients using the thoracoacromial or subclavian artery and vein grafts. Although thoracodorsal vessels are discussed as recipient vessels, we believe that the internal mammary artery and vein are the gold standard for autologous breast reconstruction (38–41). The main reasons are the easy preparation of the internal mammary vessels, their good blood flow and diameter, and the preservation of the latissimus dorsi in case of required secondary reconstruction upon free flap failure.

Originating from the TRAM flap, equally whether the pedicled or free flap version, abdominal flaps for breast reconstruction experienced a consequent further development (3, 4, 42). In this regard, Koshima and Soeda (8) introduced the DIEP flap, whereas Nahabedian et al. (43) popularized the muscle-sparing TRAM. The latter ones preserve the anterior rectus sheath, especially (parts of) the rectus muscle with its remaining laterally based innervation and blood supply. Both components, the anterior rectus sheath and the remaining neurovascular supply, play a major role in abdominal wall stabilization after flap harvest (44, 45). In the literature, hernia rates of approximately 10% for pedicled TRAM (range, 0–21.1%), 6% for free TRAM, 2% for ms-TRAM (range, 0–5%), and 3% for DIEP flaps (range, 0–7.1%) were found (46–50). In our study, we found four abdominal hernias (2%), which is comparable to the pertinent literature. Nevertheless, one has to bear in mind that not all surgeons perform anterior rectus sheath reconstruction in the same manner, especially with mesh materials. Besides rectus sheath reconstruction, preoperative CTA can help to preserve the remaining lateral abdominal wall perfusion (51). Taken together, the combination of preoperative CTA and anterior rectus sheath reconstruction may reduce abdominal hernia (47, 52, 53). In the rare event of a true postoperative hernia, we advocate abdominal wall reconstruction together with hernia surgeons.

Besides the clinical evaluation of the flap perfusion, we performed intraoperative fluorescence angiography. The routine use of this imaging tool and early adoption of this technique in a university setting may be an explanation for the excellent performance and the high success rate despite the various surgeons and their individual learning curves (54).

From our point of view, intraoperative fluorescence angiography helps to objectively assess flap perfusion and individually tailor the optimally perfused tissue parts (10, 11, 55). Consequently, insufficiently perfused flap parts can safely be discarded right away. This limits and reduces the rate of postoperative skin and fat necrosis or wound healing disorders. As most of the abdominal free flaps were performed by three senior surgeons, one has to bear in mind that always two to three residents were involved in the operation. The residents prepared the recipient vessels and assisted during the flap harvest and anastomosis, as well as rectus sheath/abdominal closure. Regardless the heterogeneous education year of the residents (range, 1–6 years), we did not observe any statistical difference of the operation time.

Although this is a retrospective single-center study, our results and the pertinent literature prove that autologous breast reconstruction, using abdominal free flaps, is a safe procedure in high-volume microsurgery centers, even following a previous radiation and regardless of patient's age (42, 56–58). Preoperative CTA visualizes abdominal wall vasculature, thereby minimizing operation time and morbidity. In case of arterial reconstruction, one has to bear in mind an increased thrombosis and consequently flap loss rate. However, the interdisciplinary approach together with radiologists, gynecologists, and general and vascular surgeons ensures the success in complex cases.

## Data Availability Statement

The datasets generated for this study are available on request to the corresponding author.

## Ethics Statement

The studies involving human participants were reviewed and approved by ethical review committee of the Friedrich-Alexander-University of Erlangen-Nuremberg (AZ 291_19 Bc). Written informed consent for participation was not required for this study in accordance with the national legislation and the institutional requirements.

## Author Contributions

DS, RH, and AA made substantial contributions to the study conception and design. DS, RH, AA, IL, JB, and MS made primary contributions to acquisition of data, analysis and interpretation. All authors participated in drafting or revising the article for important intellectual content and gave final approval of the manuscript.

### Conflict of Interest

The authors declare that the research was conducted in the absence of any commercial or financial relationships that could be construed as a potential conflict of interest.
